# Telemedicine-based system for quality management and peer review in radiology

**DOI:** 10.1007/s13244-018-0629-y

**Published:** 2018-05-18

**Authors:** Sergey Morozov, Ekaterina Guseva, Natalya Ledikhova, Anton Vladzymyrskyy, Dmitry Safronov

**Affiliations:** Research and Practical Center of Medical Radiology, Department of Health Care of Moscow, 28-1, ul. Srednyaya Kalitnikovskaya, Moscow, 109029 Russia

**Keywords:** Teleradiology, Peer review, Quality, Radiology, Diagnostic errors

## Abstract

**Objectives:**

Quality assurance is the key component of modern radiology. A telemedicine-based quality assurance system helps to overcome the “scoring” approach and makes the quality control more accessible and objective.

**Methods:**

A concept for quality assurance in radiology is developed. Its realization is a set of strategies, actions, and tools. The latter is based on telemedicine-based peer review of 23,199 computed tomography (CT) and magnetic resonance imaging (MRI) images.

**Results:**

The conception of the system for quality management in radiology represents a chain of actions: “discrepancies evaluation – routine support – quality improvement activity – discrepancies evaluation”. It is realized by an audit methodology, telemedicine, elearning, and other technologies. After a year of systemic telemedicine-based peer reviews, the authors have estimated that clinically significant discrepancies were detected in 6% of all cases, while clinically insignificant ones were found in 19% of cases. Most often, problems appear in musculoskeletal records; 80% of the examinations have diagnostic or technical imperfections. The presence of routine telemedicine support and personalized elearning allowed improving the diagnostics quality. The level of discrepancies has decreased significantly (*p* < 0.05).

**Conclusion:**

The telemedicine-based peer review system allows improving radiology departments’ network effectiveness.

**Main Messages:**

• *“Scoring” approach to radiologists’ performance assessment must be changed.*

• *Telemedicine peer review and personalized elearning significantly decrease the number of discrepancies.*

• *Teleradiology allows linking all primary-level hospitals to a common peer review network.*

## Introduction

The progress of communication and diagnostic technologies has created the ability to collect images in one place, transmit them over a distance via protected digital lines, and view them remotely for diagnostic or consultative purposes. This form of distant professional collaboration is commonly known as teleradiology.

Teleradiology has quite an interesting history, which begun in the 1920s with very simple telecommunications [12]. Nowadays, telemedicine is embedded into the workflows of many radiology practices in the global prospect. It allows to improve the work efficiency and to overcome the lack of staff and expertise [8, 10]. The clinical and economic benefits of teleradiology are well proven [2, 5, 13, 15, 16]. So, does any evolution for teleradiology exist? We believe that the answer is positive. Further development of teleradiology is linked with healthcare management.

Nowadays, the problems of quality control have become more and more significant and expensive, not only for the radiology itself, but for healthcare systems as a whole. The internal check (peer review) is an obligatory component of any radiological service today. Peer review is any method by which radiologists are reviewing their colleagues’ cases for a variety of purposes, such as credentialing, re-credentialing, and/or quality checks. In the USA, Joint Commission International (JCI) requires a departmental peer review for the accreditation of hospital radiology departments. Therefore, almost all radiology departments, at least in academic institutions, participated in a form of peer review process. The radiology department or the parent health system is free to decide how this peer review is performed [14]. The goal of the peer review in radiology is to improve the overall performance by recognizing unperceived findings on diagnostic studies and identifying opportunities for improvement.

The problem of peer review in radiology is that, most often, this is an internal procedure. This affects both the objectivity of the quality control and its accessibility, especially in primary-level hospitals with limited personnel. In sum, current peer review models are usually focusing on the “scoring” of errors and not on their “elimination”. In addition, the “scoring” approach may create tension between radiologists [2, 6].

Our team believes that the future of teleradiology is the distant peer review process, as this is the perfect tool for healthcare improvement. This approach allows increasing quality control and objectivity. On the other hand, not only a telemedicine peer review but also a system of linked actions for quality assurance in radiology should be developed. Such a system has to replace the “scoring” approach with more advanced and effective strategies.

The objective of this paper was to create and evaluate the efficiency of a telemedicine-based quality assurance system in radiology.

## Materials and methods

A concept and system for quality assurance in radiology was developed. The concept was realized at the beginning of 2016 as a set of strategies, actions, and tools.

For a period of one year (August 2016 to September 2017), all computed tomography (CT) and magnetic resonance imaging (MRI) images, performed in municipal outpatient hospitals of Moscow, were uploaded to the regional radiological system. From this general sample (*n* = 380,515), a set of studies (*n* = 23,199) was randomly selected and directed for a peer review. Prior to the peer review, personal data were removed, thus ensuring the anonymity of the patients. A group of experts, two or three for each record, performed the distant peer review. The logistics of the peer review consists of several iterations. If one of the experts considers that the discrepancy is significant, the system sends the study to the another expert. In case that the second expert disagrees with the conclusions of his colleague, the study is redirected for final evaluation to the third expert of the same subspecialty.

The quality control focuses on:

- Technical performance: artifacts, selection of the study region, patient’s positioning, scanning technique, contrast enhancement timing and phases, pulse sequences, etc.
- Diagnostic performance: detection of pathology, discrepancies in the interpretation, terminological errors, etc.

The scoring consists of four grades:

1. No discrepancy;
2. General remarks: comments on terminology, protocol design, etc.;
3. Discrepancy insignificant from clinical point of view, not affecting the treatment and/or the quality of life;
4. Discrepancy significant from clinical point of view, hypothetically affecting the treatment and/or the quality of life.

The outcomes were assessed comparing the levels of the significant/insignificant discrepancies during the first and last quarters of the study period.

The descriptive statistics included means and standard deviations. Since the data were normally distributed (Shapiro–Wilk test), the parametric *t*-test was used for the analysis. The level of significance was set at *p* < 0.05. Statistical analysis was performed with MedCalc® software.

## Results

The theoretical concept of a telemedicine-based peer review represents a cycle of actions that may be presented as a chain: “discrepancies evaluation – routine support – quality improvement activity – discrepancies evaluation”. In a nutshell, it reflects the quality improvement PDCA (plan-do-check-act) cycle. In a few words:

- The evaluation is based on an independent blinded peer review method and a formal classification of discrepancies.
- The routine support includes teleconsultations by subspecialized radiologists and technical support.
- The quality improvement activity involves different types of elearning, such as web courses, webinars, online workshops, etc., with personal learning strategies. It can also include some administrative actions, but only in especially difficult and vague clinical cases.

When the methodology just described is combined with a telemedicine network, a new tool for quality management in radiology is available. The authors succeeded in turning the theory into practice.

In 2015, the Unified Radiological Information Service (URIS) was created and launched. This is a radiological information system which unites 75 outpatient municipal hospitals, plus the Expert and eLearning Center, which was established at the Research and Practical Center of Medical Radiology, Department of Health Care of Moscow. The URIS combines 62 CT, 40 MRI, 30 digital mammography units, and approximately 400 radiologists and technicians. At the time of the preparation of the manuscript, more than a million studies and their reports have been uploaded to the system.

Most important is that the URIS is not an archive of medical images. Instead, it is a telemedicine network, with a distributed archive, with an implemented workflow and quality improvement cycle.

Every CT or MRI image or digital mammography arrives at the URIS. Each radiologist may submit a case for teleconsultation to the Expert and eLearning Center. The only requirement is to follow the preliminary distributed guidelines. The efficiency of the telemedicine consultation has been published elsewhere.

The URIS also allows monitoring the equipment parameters (loads, functions, protocols, doses) via a digital dashboard. Hence, the technicians can receive recommendations about the equipment settings if and when necessary. All of these actions are part of the daily routine support provided by the URIS.

Approximately 7% of all studies are randomly selected and sent for peer review. When systemic discrepancies are detected, a personal learning strategy is developed. The latter can be focused either on the departmental management or on a physician or a technician.

We have had this workflow in place within the URIS since December 2016. During this period 23,199 studies were randomly selected for peer review.

Clinically significant discrepancies were detected in 6% of all cases during the research period of 12 months. Clinically insignificant discrepancies were found in 19% of the cases. Most frequently, discrepancies have been revealed in the reporting of pancreas (28%), lymph nodes and peritoneum (18%), anterior abdominal wall (18%), liver (12%), pelvis (12%), and intestine (9%). The frequency of discrepancies was different in various diagnostic groups. Most often, problems appeared in oncology (46%; clinically significant - 9%, insignificant - 37%), infections (32%; 7% and 27%, respectively), and cardiovascular examinations (24%; 5% and 19%, respectively). The worst situation was for musculoskeletal imaging; almost 80% of studies had technical or diagnostic discrepancies. The highest level of diagnostic imperfections was detected in case of trauma MRI (70%). At the same time, there were only 26% of trauma CTs with discrepancies.

The detection of the technical deficits during the peer review and the special support of technicians is very important. The technical aspects of the examination critically influence the radiologists’ decision-making. A moderate correlation between the discrepancies and inadequate technique (correlation coefficient = 0.5, *p* < 0.05) or artifacts (correlation coefficient = 0.3, *p* < 0.05) for trauma cases was revealed. The technical problems most often detected were for pelvis MRI (55%), i.e., patient and slice positioning (43%), field-of-view selection (23%), and pulse sequence selection (19%) For CT, the most problematic areas are the neck and the larynx. Approximately 42% of studies were performed with technical imperfections.

The detected problems and methods of their future prevention were described in more than 220 elearning activities (including 27 web courses for 1955 radiologists, 98 webinars, 82 workshops, etc.). The regular broadcasting and free access to the records have made our webinars very popular in the professional environment. In 2017, 10,200 students from 20 regions of Russia and Commonwealth of Independent States (CIS) countries also took part in the above elearning activities.

**Fig. 1:**
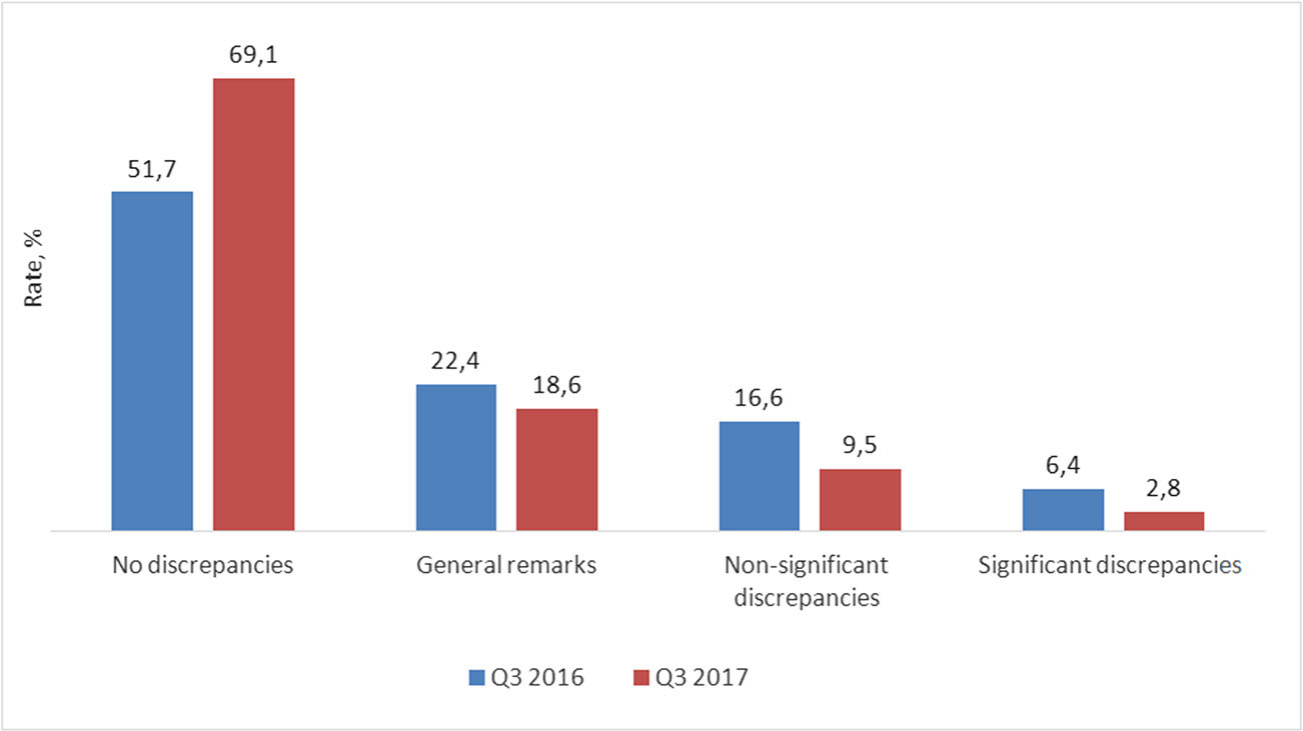
The effectiveness of a telemedicine-based quality assurance system: the rate (%) of the discrepancies and the correct radiologic reports before (Q3 2016) and after (Q3 2017) the implementation of the system

The presence of routine telemedicine support and an elearning system allowed improvement of the quality of the diagnostics. One year after the establishment of the URIS, a valid decrease of imperfections (*p* < 0.05) was observed. The level of clinically significant discrepancies decreased from 6.4 ± 2.9% (64) to 2.8 ± 0.8% (104). The number of general remarks remained roughly the same [22.4 ± 3.0% (226) vs. 18.6 ± 1.59% (691)] (Fig. 1).

The presence of the technical and medical data from the city hospitals’ network allows detecting and eliminating systemic diagnostic problems. For example, at the beginning of the research period, it was found that 66% of the outpatient examinations were performed without intravenous contrast enhancement. There was a correlation between the inadequate scanning parameters (CT without contrast enhancement) and the diagnostic discrepancies in cancer patients (correlation coefficient = 0.6, *p* < 0.05). This result invoked a special strategy focused on learning, information sharing, and general awareness about contrast enhancement. The number of outpatient CTs with contrast enhancement increased by a factor of 2.1 in a 6-month period due to the realized strategy.

Finally, the authors discovered that 90% of studies with imperfections belong to a limited group of specialists: 11% of radiologists and 17% of technicians. This result allowed to personalize and focus the learning strategies.

## Discussion

In the context of radiology quality performance evaluation via telemedicine, the results presented here are in parallel with another research [1]. A study with dual reporting was conducted in a parallel reading environment in a teleradiology practice for 3779 radiological procedures, performed at two radiology centers in the USA over a period of 4 months. The examination type was significantly related to error frequency (*p* = 0.0001), with higher than average frequencies of errors seen for CT of the abdomen and pelvis and MRI of the head and spine, but lower than average for the head and spine CT, and for ultrasound [1]. Otherwise, there is a majority of discrepancies related to the head CTs. Comparison of the conventional approach and the teleradiology system allows detecting major discrepancies in 5.8% of cases and minor discrepancies in 21.1% of the cases [7]. Differences in the classifications of the discrepancies create barriers for an objective comparison. The authors may have only observed the parallels in the higher levels of imperfections for the neck CTs.

Our telemedicine-based peer review has been introduced for quality improvement in the whole network of municipal outpatient hospitals. It was clarified that physician errors, discovered in the peer review process, should be used not for punishment, but for the life-long education of the radiologist, perhaps in the form of challenging/missed cases or morbidity–mortality conferences and virtual webinars [14].

Recently published papers on the peer review experience identify important opportunities to create a non-punitive peer review system, truly focused on learning from the errors we all make [9]. It was mentioned that radiologists’ reporting performance cannot be perfect and some errors are inevitable. The peer review system should create strategies to minimize errors and to learn from them [2]. The system of quality control in radiology should progress beyond the counting of errors and move on to group learning and error prevention [11]. The authors firmly believe that their approach, concept, and results closely correspond with the above-mentioned ideas. The results have already proved this.

Moreover, previous researches have demonstrated a numeric scoring of discrepancies. Our research aims to demonstrate how the quality performance evaluation positively influences radiologists’ and technicians’ skills and affects the healthcare system in general. Due to the combination of technology, learning, and management, the authors have successfully achieved a “transition from a peer-review to a peer-learning approach” [3] in radiology.

No doubt, the presented study has some limitations. The most serious one is the absence of an internationally recognized system of classifying the discrepancies and imperfections in radiology. Such a system has to be developed in further researches. Another limitation is the sample size for the regular audit. There is a theory that valuable information could be obtained when sampling at least 2.5% of each radiologist’s volume, with a maximum of 300 cases [4]. However, the authors believe that the effective sample size should be clarified in further studies. Finally, there are limitations in the comparison with other publications, due to the limited list of modalities available in the URIS as present.

## Conclusion

The “scoring” approach to a radiologist’s performance assessment is replaced by a more sophisticated evaluation method. The distant peer review process is applied for a systemic imperfections detection. Quality improvement strategies are developed. The latter unite different actions (learning, management, etc.) and are specially developed for the concrete radiology department. Thus, quality improvement procedures become more personal and more effective.

The new approach has allowed improvement of the diagnostics quality. The rate of discrepancies has decreased significantly (*p* < 0.05) after one year following a systemic telemedicine-based peer review.

Future research will be devoted to the standardization of the peer review methodology and to the development of “big data” tools for the monitoring and management of a radiology service.

## Abbreviations

CT: Computed tomography
MRI: Magnetic resonance imaging
URIS: Unified Radiological Information Service
